# Epithelial Cell Adhesion Molecule: An Anchor to Isolate Clinically Relevant Circulating Tumor Cells

**DOI:** 10.3390/cells9081836

**Published:** 2020-08-05

**Authors:** Zahra Eslami-S, Luis Enrique Cortés-Hernández, Catherine Alix-Panabières

**Affiliations:** Laboratory of Rare Human Circulating Cells (LCCRH), University Medical Centre of Montpellier, 34295 Montpellier, France; z-eslami-samarin@chu-montpellier.fr (Z.E.-S.); le-cortes-hernandez@chu-montpellier.fr (L.E.C.-H.)

**Keywords:** circulating tumor cells, epithelial cell adhesion molecule, epithelial cancer, epithelial-to-mesenchymal transition

## Abstract

In the last few decades, the epithelial cell adhesion molecule (EpCAM) has received increased attention as the main membrane marker used in many enrichment technologies to isolate circulating tumor cells (CTCs). Although there has been a great deal of progress in the implementation of EpCAM-based CTC detection technologies in medical settings, several issues continue to limit their clinical utility. The biology of EpCAM and its role are not completely understood but evidence suggests that the expression of this epithelial cell-surface protein is crucial for metastasis-competent CTCs and may not be lost completely during the epithelial-to-mesenchymal transition. In this review, we summarize the most significant advantages and disadvantages of using EpCAM as a marker for CTC enrichment and its potential biological role in the metastatic cascade.

## 1. Introduction

Cancer diagnosis and management represent a huge challenge for clinicians worldwide. The high mortality of cancer is often related to its late diagnosis and the appearance of resistance to the currently used therapies [1,2,3]. Despite significant advances in oncology, there are still limitations in screening and treating patients with carcinoma (i.e., cancer of epithelial origin). One of the best possible solutions is the identification of reliable protein biomarkers that are strongly associated with the disease outcome and that can be used for early detection, prognosis and prediction of the therapeutic response [4].

The epithelial cell adhesion molecule (EpCAM) is a transmembrane glycoprotein that has received increased attention as a “universal” tumor marker for epithelial-derived cancer types [5]. Forty years ago, EpCAM was first described as a major epithelial carcinoma antigen that is recognized by monoclonal antibodies that bind specifically to human colorectal carcinoma cells [6]. It was first considered to be an adhesion molecule, but its role in various biological functions has been progressively identified: (i) gene regulation, (ii) cell proliferation, (iii) cancer stemness, and (iv) interaction with cell adhesion molecules [7,8,9]. EpCAM is also overexpressed on the surface of the majority of primary and metastatic cancers [10,11]. Therefore, EpCAM might be a valuable marker in patients with solid cancer. Additionally, its interaction with other proteins might provide a therapeutic window to repress its growth-promoting signaling in cancer [5].

EpCAM has received considerable attention in the liquid biopsy field because it is used in the CellSearch^®^ system (Menarini-Silicon Biosystems, Italy, 2020, and Janssen Diagnostics Raritan, NJ, USA, 2004) to detect circulating tumor cells (CTCs) [12,13]. During cancer dissemination, tumor cell motility and invasiveness increase, which enables the dissociation and extravasation of tumor cells into circulation to become CTCs. Eventually, the most aggressive CTCs, which can survive in the bloodstream, will reach distant organs and form metastases. As many cancer-related deaths are the result of late diagnosis and the development of metastasis [1,2,3], the in-depth characterization of CTCs can give important information about the tumor’s molecular profile and this provides a tremendous opportunity to identify the mechanisms underlying metastasis. It could also have clinical significance. Indeed, CTC detection has been widely investigated as a tool to detect several cancer types. Moreover, the expression of various prognostic markers by CTCs has been exploited to evaluate cancer aggressiveness and patient’s overall survival (OS) [14].

As most cancers are of epithelial origin, targeting epithelial antigens was the first approach to distinguish CTCs among the millions of normal blood cells that have a mesenchymal phenotype. EpCAM quickly became the most used epithelial marker for CTC tracing and isolation in the bloodstream. However, the clinical utility of CTCs remains to be established due to their rarity and heterogeneity, and the lack of accurate methods to detect and select CTCs among the millions of other blood cells. 

In this review, we describe EpCAM biology and its role in the metastatic cascade and in targeted therapies in cancer. We then discuss the strengths and limitations of using EpCAM for CTC capture and isolation, especially in clinical studies.

## 2. EpCAM in Cancer

EpCAM is a cell-surface transmembrane glycoprotein that is expressed in healthy human epithelial tissues but also in epithelial cancers, cancer stem cells, and inflammatory diseases [15,16,17,18,19,20]. *EPCAM*, the gene encoding EpCAM, has nine exons and is located on chromosome 2 [21]. The mature form of this protein consists of a large N-terminal extracellular domain (EpEX), a single spanning transmembrane domain (TM) and a short C-terminal cytoplasmic domain (EpICD). EpEX includes the N-Domain that contains epithelial growth factor sites, the thyroglobulin type 1A domain (TY-domain), and the C-Domain. EpEX forms heart-shaped dimers on the cell surface [22]. 

EpCAM signaling requires regulated intramembrane proteolysis (RIP), a conserved signal-transducing mechanism that allows the transit of information across cellular compartments [23]. EpCAM, as a substrate of RIP, is first cleaved by metalloprotease tumor necrosis factor-alpha converting enzyme (TACE/ADAM17), which leads to the release of EpEX [24]. Then, a protease component of the γ-secretase complex presenilin 2 (PS-2) cleaves EpICD [24]. EpICD is translocated from the cytoplasm to the nucleus where it may be implicated in the Wnt pathway through involvement in a nuclear complex with lymphoid enhancer-binding factor 1 (LEF-1), four and a half LIM domain protein 2 (FHL2), and β-catenin [7,25,26,27]. This nuclear complex binds to promoters of genes that are involved in the cell cycle and stemness regulation, and can increase cancer cell proliferation [7,24,25]. Several studies have focused on the role of the different EpCAM domains in cancer. For instance, an analysis of the localization and expression of EpEX, EpICD and β-catenin in surgical specimens of extrahepatic cholangiocarcinoma indicated concomitant nuclear expression of EpICD and β-catenin [28]. Moreover, EpICD accumulation in the nucleus predicts an aggressive clinical course in patients with early stage breast cancer [29]. Likewise, analysis of colorectal and thyroid cancer samples showed that EpICD accumulation in the nucleus is strongly correlated with poor prognosis [28,30]. Besides EpICD, loss of EpEX membrane expression has been correlated with lower OS in patients with aggressive thyroid cancer [28]. 

EpCAM expression varies according to the tumor type. Indeed, it is often strongly expressed in breast, lung, colon, intestine and prostate carcinoma, whereas it is not detected in lymphoma, melanoma, sarcoma and neurogenic tumors [31]. Its expression is also different in primary and metastatic tumors [11]. Moreover, the prognostic value of EpCAM expression might be different depending on the type of cancer. For instance, higher EpCAM expression is linked to a lower median OS in patients with gastric cancer [32]. In contrast, EpCAM has a positive prognostic value in patients with head and neck squamous cell carcinoma, where it was correlated with longer OS [33]. In breast cancer, EpCAM expression was correlated with favorable prognosis in the HER2 molecular subtype, and with unfavorable prognosis in the basal-like and luminal molecular subtypes [34]. Therefore, the prognostic value of EpCAM expression is intrinsically associated with the cancer type and/or subtype.

EpCAM is involved in the regulation of cancer cell adhesion, proliferation, migration, invasion, stemness, and epithelial-to-mesenchymal transition (EMT) during cancer progression [35,36]. EpCAM expression was correlated with abnormal cell proliferation in cervical squamous epithelium for the first time in 1996 [37]. Other studies then showed the role of EpCAM in the regulation of cancer cell proliferation, migration, and invasion [38,39]. Specifically, EpCAM expression is positively correlated with the proliferation marker Ki67, the high expression and nuclear localization of cyclin D1, and Rb phosphorylation. These findings strongly suggest that EpCAM promotes cell cycle progression via the classical cyclin-regulated pathway [27]. Moreover, Gaiserit et al. generated conditional knockout mice with EpCAM-deficient Langerhans cells (LCs) to show that EpCAM promotes epidermal LC motility and migration. In esophagus cancer, high EpCAM expression has been correlated with proliferative stages, whereas low or negative expression was associated with cancer cell migration, invasion and dissemination [40]. Similarly, *EPCAM* silencing in breast cancer cell lines leads to a 35–80% reduction in the rate of cell proliferation [39]. Moreover, wild type p53 controls breast cancer invasion partly by negatively regulating EpCAM expression through binding to a response element within the *EPCAM* gene (intron 4). These studies show EpCAM’s key role in cancer development and progression. 

Although EpCAM has not been directly associated with any classical junctional structure, it interacts with different adhesion proteins and this might contribute to its role in cancer progression [41]. For instance, EpCAM modulates tight junction functions by regulating the intracellular localization and degradation of claudins (tight junction proteins) through the direct interaction of its TM domain with claudin-7 [42]. The interaction of E-cadherin, integrin αvβ6 and EpCAM on cancer cells can trigger the activation of tumor-mediated fibroblasts that then influence gene expression and sensitivity to therapeutic agents [43]. EpCAM also can inhibit cadherin-mediated cell–cell adhesion in breast epithelial cells through interaction with phosphoinositide 3-kinase [44]. Additionally, by disrupting the link between α-catenin and F-actin, EpCAM can modulate the strength of E-cadherin-mediated cell-cell adhesion [45].

The influence of EMT on EpCAM expression is still not well understood. Jojovi et al. were the first to describe the loss of EpCAM expression during EMT by immunohistochemical analysis of breast, colon, ovarian and lung tumor cell xenografts and metastases from severe combined immunodeficient mice [46]. Specifically, they found transient EpCAM downregulation in the early stages of migration. Additional studies showed that EpCAM downregulation is associated with mesenchymal features [47,48]. To determine the underlying mechanism, Sankpal et al. induced EMT in normal epithelial and epithelial cancer cell lines by incubation with cytokines (transforming growth factor-β1 [TGFβ1] and tumor necrosis factor-α [TNFα]) and found that EpCAM expression was reduced. They also showed that this effect was mediated by ERK, a key EMT regulator whose expression is regulated by EpCAM, in a double negative feedback loop [49]. Pan et al. showed that EpCAM might also activate epidermal growth factor receptor (EGFR) via its EpEX domain. They suggested another feedback loop in which EpEX binding to EGFR activates ERK2 and phosphorylation of AKT, thus promoting EGFR-dependent cell proliferation and suppressing EGF-dependent EMT [33]. Interestingly, EGF/EGFR signal transduction triggers cell-surface EpCAM cleavage, leading to nuclear internalization of its EpICD, which activates genes involved in oncogenic functions, particularly EMT. This mechanism was blocked by treatment with an inhibitor of γ-secretase that normally regulates EpCAM intra-membrane proteolysis and results in EpEX shedding from the cell surface and EpICD release in the cytoplasm [50]. This finding might explain the contradictory effects of EpCAM on proliferation/invasion and shed light on EpCAM-based plasticity in cancer progression. However, several studies do not support the finding of a direct effect of γ-secretase inhibition on EGF/EGFR–mediated EpEX shedding [51]. These examples strongly suggest that EpCAM expression changes during EMT, although other studies suggest that EpCAM is upregulated and/or promotes EMT [52,53]. 

Finally, EpCAM is not only present on the surface of cells, but also in extracellular vesicles (EVs), such as exosomes. Therefore, EpCAM can be detected in CTCs and also in circulating exosomes isolated from the blood of patients with cancer [54]. Indeed, in the liquid biopsy field, antibodies against EpCAM are among the strategies used to detect and isolate exosomes for downstream analyses [55,56]. For instance, EpCAM^+^ exosome level is associated with the stage of ovarian cancer and its aggressiveness [57]. Moreover, EpCAM has been detected in exosomes secreted by human colorectal cell-derived organoids and isolated using magnetic beads coupled to an anti-EpCAM-antibody [58]. Recently, the CellSearch^®^ system (an EpCAM-dependent method) was also applied for the enumeration of EpCAM^+^ large tumor-derived EVs (tdEVs). Nanou et al. demonstrated that a cut-off of ≥20 EpCAM^+^ tdEVs/7.5 mL in blood from patients with different cancer types can predict OS, with a prognostic value equivalent to CTC enumeration [59].

## 3. Advantages of EpCAM Use as a CTC Diagnostic Marker

**Figure 1:**
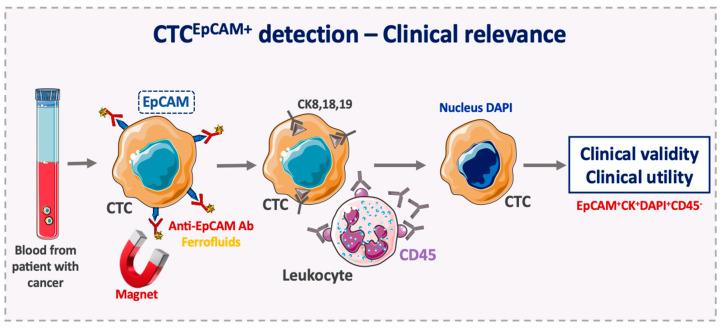
Use of the epithelial cell adhesion molecule (EpCAM) to detect clinically relevant circulating tumor cells. In the FDA-cleared CellSearch^®^ system, circulating tumor cells (CTCs) are enriched positively using ferrofluid nanoparticles coated with anti-EpCAM antibodies that bind to EpCAM-positive CTCs. Then, CTCs are selected using anti-CK8, -CK18, and -CK19 antibodies and DAPI nuclear staining. Leukocytes are excluded using anti-CD45 antibodies. CTC, circulating tumor cell; CK, cytokeratin; Ab, antibody; EpCAM, epithelial cell adhesion molecule.

EpCAM-based enrichment for CTC detection has provided a reliable prognostic tool in different cancers. The CellSearch^®^ system, the only US Food and Drug Administration (FDA) approved system and currently the gold standard for CTC detection, is the most widely used technology for prognostic purposes in clinical studies. It is based on the enumeration of epithelial cells that are separated from whole blood samples by positive enrichment using anti-EpCAM antibodies coated with magnetic beads (Figure 1) [13,60]. Specifically, the ferrofluid reagent consists of particles with a magnetic core surrounded by a polymeric layer coated with anti-EpCAM antibodies for directly capturing CTCs. After immunomagnetic capture and enrichment, fluorescent antibodies (against cytokeratin, CK, 8,18 and 19 that are expressed in the CTC cytoplasm; and against CD45 expressed on the leukocyte surface) and the nuclear dye DAPI are added for CTC identification and enumeration. Thus, CTCs are defined as EpCAM^+^/CK8/18/19^+^/DAPI^+^/CD45^−^ cells [12]. Overall, this EpCAM-based enrichment step allows a relatively pure sample to be obtained. However, EpCAM^low/negative^ CTCs are not detected with this system, thus CTC recovery rate might not be optimal, which results in an underestimation of CTC number.

The first two studies [9,15] that used this EpCAM-based enrichment method represent the beginning of a still growing number of published studies that show evidence for the relevance of CTC enumeration. In 2004, Cristofanilli et al. used CTC enumeration to evaluate cancer progression and survival of patients with metastatic breast cancer before and after treatment. They found that progression-free survival (PFS) and OS were shorter in patients with ≥5 CTCs per 7.5 mL of whole blood compared with patients with <5 CTCs. Moreover, the reduction in the percentage of patients with ≥5 CTCs at the first follow-up visit after treatment initiation suggested that the therapy was beneficial. [12]. Simultaneously, Allard et al. showed the analytical accuracy, sensitivity and reproducibility of CTC detection with the CellSearch^®^ system by using blood samples from healthy controls and patients with benign and metastatic carcinoma. Although CTC detection was variable among cancer types, CTCs were extremely rare or absent in controls [61]. Then, De Bono et al. in a prospective study on patients with castration-resistant prostate cancer demonstrated that CTCs are the most accurate and independent predictor of OS, and that CTC counts predict OS better than PSA decrement algorithms at all time points [62]. In another study on metastatic colorectal cancer, patients were stratified in two groups (unfavorable and favorable prognosis) based on a cut-off of ≥3 CTCs per 7.5 mL of blood. This cut-off was a good predictor of OS and PFS. Baseline and follow-up CTC levels remained strong predictors of PFS and OS after adjustment for clinically significant factors [63]. The data obtained in these four clinical trials were used by the FDA to approve the CellSearch^®^ system for CTC enumeration.

Therefore, targeting EpCAM to capture CTCs is a relevant approach and it demonstrates the clinical relevance of CTCs. Indeed, strong associations between reduced CTC count and PFS or OS have been established, and the change from high to low CTC count after therapy indicates good prognosis in breast [12,64], prostate [62,65], and colon cancer [63]. More recently, CTC analysis has been proposed for many clinical applications [66] including (i) evaluating the risk of metastatic relapse (prognosis), (ii) real-time monitoring of the treatment response, (iii) identification of therapeutic targets and resistance mechanisms, (iv) patient stratification and therapeutic intervention, and (v) screening and early detection of cancer. Finally, detecting EpCAM^+^ CTCs with the CellSearch^®^ system is important because their presence is always correlated with the clinical outcome.

Besides the CellSearch^®^ system, many other EpCAM-based methods have been developed for CTC enrichment, capture and enumeration, such as the MagSweeper, an EpCAM-based immunomagnetic separation method [67]; the GILPUI CellCollector^®^ for the in vivo capture of EpCAM^+^ CTCs using a nanowire in the arm vein for 30 min [68]; the IsoFlux that combines EpCAM-coated magnetic beads with microfluidic processing [69]; and the Microvortex-Generating Herringbone-Chip, a microfluidic device with EpCAM-coated microposts [70]. However, all of these methods still require analytical and clinical validation, and to date, none have been cleared by the FDA.

The EpCAM-independent integrated subtraction enrichment and immunostaining-FISH (SE-iFISH) method was used to investigate the role of EpCAM expression on CTCs and disseminated tumor cells (DTCs) in patients with breast cancer. Among the isolated CTCs, EpCAM^+^ CTCs were only detected in patients with metastatic cancer. Moreover, the mean DTC number per patient was six times higher and the percentage of EpCAM^+^ DTCs was significantly higher in patients with metastatic cancer than in patients without metastases (66.53% vs. 8%). This suggests that EpCAM^+^ CTCs and DTCs could be reliable biomarkers for evaluating therapeutic efficacy and predicting cancer prognosis [71]. 

A study on esophageal cancer found that the majority of DTCs in bone marrow lacked EpCAM expression, while EpCAM was strongly expressed in the tumor [40]. EpCAM downregulation was also associated with partial loss of the epithelial phenotype. The authors showed that natural or experimental (knock down) loss of EpCAM reduced the proliferation rate, but promoted cancer cell migration and invasion [40]. Additionally, the number of EpCAM^+^ DTCs was correlated with significantly lower OS in patients with esophageal cancer [40]. High EpCAM expression is associated with proliferation, while EpCAM^low/negative^ expression is correlated with migration, invasion and tumor cell dissemination. Therefore, the EpCAM phenotype might be a helpful guide for therapeutic decision-making and should be taken into account when analyzing DTCs [40].

## 4. Disadvantages of EpCAM Use as a CTC Diagnostic Marker

Although EpCAM-based methods allow the identification of CTCs from epithelial cancers, many CTCs detected in patients with different cancers [72,73,74] do not have sufficient epithelial characteristics (i.e., there is a lack of or low expression of EpCAM). The failure of EpCAM-based technologies to detect such CTCs might be explained by several reasons: (i) inefficiency of antibody clones that results in variable CTC capture yields [75], and (ii) phenotypic plasticity. For instance, CTCs can undergo EMT and mesenchymal-to-epithelial transition (MET). During the EMT of CTCs, some epithelial markers might be downregulated (e.g., the loss or reduced expression of epithelial cell surface markers, such as EpCAM and E-cadherin), while some mesenchymal surface markers are upregulated (e.g., N-cadherin) [76]. In vivo experiments showed that CTC detection using EpCAM-based technologies is limited by the presence of CTCs undergoing EMT [77]. EpCAM^+^ CTC detection also varies among different types of carcinoma. For instance, high numbers of EpCAM^+^ CTCs are often detected in blood samples from patients with breast, prostate and small cell lung cancer. Conversely, EpCAM^+^ CTC count is low in patients with pancreatic, colorectal and non-small cell lung cancer [78]. This could be explained by the presence of CTCs undergoing EMT, but also by the tumor’s anatomical location [79]. Both factors could limit the use of EpCAM-based technologies for CTC enrichment. Moreover, because EpCAM-based technologies for CTC detection are optimized for carcinoma, they are not appropriate for detecting CTCs derived from mesenchymal cancers (sarcoma, lymphoma, and neurogenic tumors) [19,31].

The downregulation of EpCAM in CTCs compared with primary and metastatic tumors suggests that EpCAM expression might be transient and related to EMT [80]. Yu et al., evaluated the expression of epithelial and mesenchymal markers in CTCs from patients with metastatic breast cancer by using an EpCAM-independent isolation method. This demonstrated CTC heterogeneity as EMT markers were differently expressed in CTCs from different breast cancer subtypes. Moreover, in CTCs from patients with progressive disease after chemotherapy, they observed a change in CTC from a predominant epithelial to a mesenchymal phenotype, suggesting that EMT plays a role in treatment resistance [81]. Other groups also reported the association between EMT and chemoresistance [82,83]. For instance, Fischer et al. showed that EMT is associated with cyclophosphamide resistance using an in vivo model of metastatic breast cancer and fibroblast specific protein 1 (Fsp1) as an EMT marker [82]. However, as EMT is a complex process that involves several molecular pathways, a single marker to define EMT might not represent the whole process. Indeed, most studies on EMT have similar limitations with regard to the correct definition of EMT and MET [84]. Therefore, it is not possible to make a firm conclusion about the role of EMT in chemoresistance. This complexity is also a reflection of the heterogeneity of CTCs.

Alternative methods, such as surface-enhanced Raman spectroscopy-based biosensors, have been suggested to trace EpCAM expression in cancer cells during EMT [45], but they have not been tested for CTC detection yet.

Therefore, the scientific community has raised doubts on whether EpCAM-based methods are appropriate to detect all the CTCs that are relevant to metastatic progression or to therapeutic resistance. Many groups are trying to improve the existing technologies or to develop new systems for CTC enrichment and isolation [85] by focusing mainly on EpCAM-independent CTC identification approaches [60,86,87,88]. Several groups have combined different markers for CTC isolation. They showed that the sensitivity of CTC detection can be increased by using EpCAM with other epithelial markers (e.g., HER2, HER3, EGFR and MUC1) [89,90,91,92] or some mesenchymal markers (e.g., vimentin, N-cadherin, twist) [93,94,95]. For instance, CTC detection in patients with metastatic colorectal cancer is improved when the CellSearch^®^ system is combined with the AdnaTest^®^ (AdnaGen GmbH, Langenhagen), which uses RT-PCR to detect *EPCAM*, *EGFR*, and *CEA* expression in the EpCAM-enriched cell fraction [96].

Other groups have developed EpCAM-independent technologies, mainly based on the physical features of CTCs (such as electrical charge, density, size and deformability). One example is the ParsortixTM PC1 system (ANGLE North America, Inc., King of Prussia, PA, USA), a microfluid device that captures CTCs based on their size and deformability. This method has the theoretical advantage of capturing CTCs with low expression of epithelial markers [97]. A clinical trial (ANG-002; NCT03427450) is currently assessing the validity and utility of this method. Another example is ISET^®^ (Isolation by Size of Tumor cells), a filtration system in which CTCs are selected as a result of their large size. ISET^®^ has already been used for CTC isolation in clinical studies [98,99]. However, EMT not only leads to a reduction in EpCAM expression, but can also affect the physical features (mass and size) of CTC [48].

## 5. EpCAM Expression on Metastasis-Competent CTCs

EpCAM is strongly expressed not only on cancer cells, but also on stem cells. For instance, in the intestinal epithelium, there is a gradient of EpCAM expression from the crypts (where stem cells are located) to the villi (where differentiated cells reside) [100]. EpCAM is also implicated in the proliferation of human embryonic stem cells, because *EPCAM* knockdown in such cells significantly reduces their proliferation rate independently of other stem cell markers [101]. Therefore, EpCAM has been proposed as a stem cell marker, and its presence in cells should be understood as a proliferative marker, and not just as an epithelial marker [25,102].

EpCAM overexpression is frequently observed in cancer tissue samples from patients with colon, stomach, prostate, kidney, ovary, liver, lung, and breast cancer [11,103,104], and correlates with poorer prognosis. This suggests that EpCAM overexpression in tumor cells might be associated with the presence of a higher number of cancer stem cells (CSCs) that can become metastatic-initiator cells (MICs).

**Figure 2:**
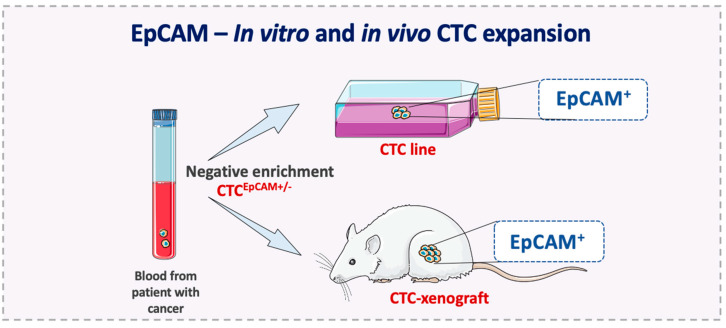
In vivo and in vitro CTC expansion. In vitro and in vivo models are essential to identify and characterize metastasis-competent CTCs. The successful expansion of this more aggressive subset of CTCs after negative selection showed a clear EpCAM-positive phenotype in different studies from independent research groups [105,106,107,108,109,112,113].

CTCs that overexpress EpCAM might represent the CSC and MIC subpopulations in the tumor. This hypothesis is supported by clinical studies showing that EpCAM^+^ CTC number is associated with prognosis [12], and by the finding that EpCAM expression is detected in all established CTC lines and in CTCs expanded for a short time [105,106,107,108,109,110,111]. For example, Yu et al. established long-term CTC lines from breast cancer by using an inertial focusing-enhanced microfluidic method for EpCAM^+^ CTC enrichment. This assay is based on hydrodynamic cell sorting from whole blood, and it is combined with immunomagnetic bead sorting (negative depletion of leukocytes with an anti-CD45 antibody, or positive enrichment of CTCs, with an anti-EpCAM antibody) [105]. Similarly, Cayrefourcq et al. obtained stable and permanent CTC lines from blood samples of patients with colorectal cancer using the Ficoll–Hypaque density gradient centrifugation method, which includes a leukocyte depletion step. The authors showed that these CTC lines can be used to develop tumor xenograft models that express EpCAM [106]. Moreover, using a xenograft assay, Bacelli et al. demonstrated that CTCs with a tumor-initiating phenotype (CD45^−^, EpCAM^+^, CD44^+^, CD47^+^, MET^+^) can produce metastases [109]. Similarly, Koch et al. established an EpCAM^+^ CTC line derived from breast cancer [112] and Faugeroux et al. generated CTC-derived explant models by using EpCAM^+^ CTCs isolated from blood samples of patients with castration-resistant prostate cancer (Figure 2) [113].

These studies suggest that EpCAM expression in CTCs is important to complete the metastatic cascade. Indeed, to establish long-term and permanent CTC lines (or CTC xenografts) these cells must have acquired the ability to grow independently, as observed in MICs. However, due to the high variety of cancer types and subtypes, metastasis formation might not always require EpCAM expression. For instance, Zhang et al. identified a subpopulation of EpCAM-CTCs in vitro and in vivo that form brain metastases and that express EGFR and human epidermal growth factor receptor 2 (HER2) [114]. These markers correspond to the phenotype of the HER2 intrinsic molecular subtype of breast cancer that, unlike luminal B and basal-like, is not associated with worse prognosis when EpCAM is expressed [34]. In other words, the heterogeneity in cancer molecular subtypes might limit the generalization of the importance of EpCAM expression and CTCs in clinical studies.

Although in vitro expansion of CTCs had been successfully achieved by using EpCAM- dependent and -independent methods [106,107,108,112,114], as mentioned above, long-term CTC culture has been possible only for EpCAM^+^ CTCs. These observations together with the association of EpCAM expression with CSCs and the prognostic role of EpCAM^+^ CTCs, strongly suggest that EpCAM-based technologies might detect CTC populations that include MICs, and that despite their limitations, these methods still offer the most practical approach for most cancer types.

**Figure 3:**
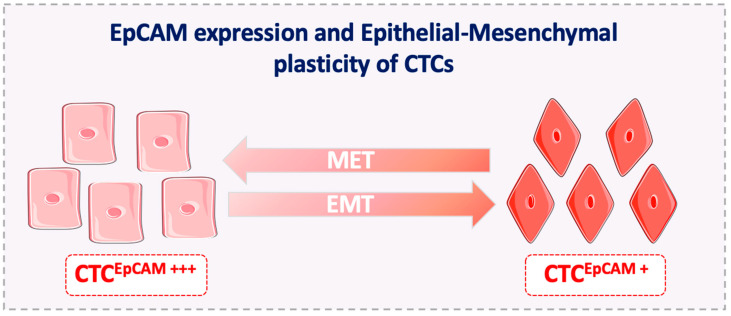
EpCAM expression and epithelial-mesenchymal plasticity of CTCs. CTCs that undergo EMT obtain an intermediate phenotype and often continue to express EpCAM. Indeed, the downregulation of EpCAM expression, if initiated, is rarely complete during the EMT in CTCs. EMT, epithelial-to-mesenchymal transition; MET, epithelial-to-mesenchymal transition.

Finally, metastasis-competent CTCs display phenotypic plasticity and can acquire migratory features. If full EMT occurs, EpCAM expression is completely lost [115], and EpCAM-based methods cannot detect this CTC subpopulation. However, it has been suggested that EMT is a gradual and reversible process (partial EMT) that is dictated by epigenetic mechanisms [84]. Therefore, CTCs that undergo partial EMT express epithelial and mesenchymal markers (partial phenotype) and this increases their survival fitness. This means that cells displaying less phenotypic plasticity will not achieve the fitness required to successfully finalize the metastatic cascade. Thus, the clinical detection of metastasis-competent EpCAM^+^ CTCs might not be limited by EMT, at least in cancer types/subtypes where EpCAM expression is strongly associated with cancer progression (Figure 3).

## 6. EpCAM^+^ CTCs Might Predict the Outcome of EpCAM-Targeted Therapies

As EpCAM is highly expressed on the surface of many cancer cells and has been associated with tumor cell proliferation, migration and invasion, some targeted therapies have been developed against this protein. For example, edrecolomab is a mouse-derived monoclonal antibody that was approved for clinical applications, based on results from early clinical trials that showed complete remission in metastatic colorectal cancer [116,117]. However, another clinical trial demonstrated that edrecolomab was in fact inferior to the standard 5-fluorouracil-based chemotherapy [118]. This resulted in its withdrawal from the market. The lack of effectiveness of this antibody could be explained by its low binding affinity to EpCAM. Adecatumumab, is another anti-EpCAM antibody that was developed to increase the binding affinity to EpCAM; however, this antibody did not show any effect (i.e., tumor regression) in patients with metastatic breast cancer [119,120]. Nevertheless, in a retrospective analysis, patients with high EpCAM expression that were treated with high doses of adecatumumab showed a lower risk of developing new metastases [119]. This observation suggests that adecatumumab might mainly target cancer cells with metastatic potential that strongly express EpCAM. These cells might be EpCAM^+^ CTCs (or EpCAM^+^ CTC precursors). The disappointing results of these trials may be partly explained by the inaccurate selection of patients. Indeed, none of these trials selected patients based on the number of EpCAM^+^ CTCs in blood. As their number in blood can vary among patients with the same cancer type [78], selecting patients based on the EpCAM^+^ CTC count in the peripheral blood could be a better and more precise way to identify patients who could benefit from such therapies. CTC enumeration has been already used to pre-select patients for specific therapies. For instance, in the STIC-METEBREAST trial on metastatic breast cancer, a cut-off of ≥5 CTCs per 7.5 mL was used to predict the failure of hormone therapy, and these patients were switched to a more aggressive treatment [121,122]. Similarly, for EpCAM targeted therapies, a higher number of EpCAM^+^ CTCs might predict the efficacy of edrecolomab or adecatumumab.

Other anti-EpCAM approaches have been developed, such as chimeric antigen receptor T-cell (CAR-T cell) therapy. However, their efficacy is limited by EpCAM expression in healthy tissues (e.g., gastrointestinal tract), which might cause undesirable secondary effects. Perhaps, the use of CTCs as a predictive marker could help to minimize these secondary effects. Specifically, routine CTC analysis during treatment might contribute to better evaluate the treatment effects, to limit the use of anti-EpCAM therapies to patients with clear benefits, and to stop the therapy once it is no longer effective.

A disadvantage of any EpCAM-targeted therapy is that it might lead to EMT induction or selection of resistant clones. Indeed, as observed with all cancer therapies, cancer cells adapt to and generate mechanism of resistance. However, EpCAM might be a key molecule for CTC colonization, and metastatic tumor formation, at least for most of the cancer types in which EpCAM^+^ CTCs are abundant. Although the relevance of EpCAM targeted therapies is not clear, EpCAM^+^ CTCs might help to select patients who would most benefit from these treatments.

## 7. Conclusions

CTCs provide more comprehensive molecular information on metastatic cancer than a single metastatic lesion because they can represent tumor heterogeneity. CTC clinical applications in various cancer types have been validated by EpCAM-based technologies. A high number of EpCAM-expressing CTCs has been correlated with reduced OS in different cancer types [61,62,63,123]. Understanding the regulation of variations in EpCAM expression in CTC subpopulations and during EMT might provide new insights on the biology of the metastatic process. EpCAM is a relevant marker for CTC detection [124], although the total CTC count is underestimated due to the presence of EpCAM-negative CTCs. For instance, the detection of CTCs in only about 60–80% of patients with metastatic breast cancer may be explained by the presence of EpCAM-negative CTCs in some patients [86,125]. As the EpCAM-based CellSearch^®^ system remains the only technology validated for CTC detection in the clinic, other markers should be included to overcome its limitations. In addition, the current methods need to be improved and new technologies developed [60] to resolve the ongoing debate and provide insights into CTC biology.

EpCAM might also be a target for personalized medicine. Therapies against EpCAM^+^ CTCs could target not only cancer cells in the primary tumor, but also CTCs as the drivers of cancer dissemination [126,127]. The potential roles of EpCAM in cell transformation, and its strong impact on the activity of metastasis-initiating cells and on the regulation of self-renewal and pluripotency in stem and progenitor cells [7] highlight its relevance in the metastatic cascade. EpCAM expression is also associated with cancer stem cell-like phenotypes in breast cancer that contribute to the formation of bone metastasis [128].

Finally, the development of CTC lines and the recent results from interventional clinical trials support the observations that EpCAM has a key role in the metastatic cascade. Moreover, EpCAM is a target marker on tumor cells, and it is also expressed on the membrane of other liquid biopsy analytes, such as EVs. Therefore, this protein will play a fundamental role in the liquid biopsy field in the future.
